# News and Notes

**Published:** 1911-07

**Authors:** 


### REGULAR MEETING FULTON COUNTY MEDICAL SOCIETY.

### APRIL 6, 1911.

REPORTED BY R. R. DALY, M. D.

Dr. Neall read a paper on "Arterio-Sclerosis, its Etiology, and Treatment."

Dr. Duncan said that alcohol, toxines, condiments, etc., enumerated are undoubtedly effective in causing the trouble; even digitalis given too frequently may bring it about. The coronary vessels show degeneration in agina pectoris. The treatment embraces proper attention to the kidneys and various other organs. Worry is a very potent cause especially among doctors who can not leave their business when they return to their homes. If tobacco will help to dispose of the worry, it is better to use it.

Dr. Kime said that one essential in the treatment is prophylaxis, and that hereditary predisposititon should be looked to. The influence of the adrenal glands should be considered because the change in their functions will bring about arterial trouble. Alcohol, tobacco, tea, coffee and excessive eating of meat will cause the disease. He agrees with Dr. Duncan in the effects of worry and the strenuous life.

Dr. Thrash said we are all too much inclined to consider what others say, not what we observe ourselves. Arterio-sclerosis is not a disease; it is the result of nature trying to give more resistance to the tissues in replacing injured ones. This injury comes from other sources. Atrophy of the intima in the blood vessels is not always dependent upon excess of alcohol. Many men who never drank have it. Worry causes faulty and improper combustion; the toxines resulting from this irritate the intima of the vessels and harm them. The real disease is present before the repair system of sclerosis appears. The subject to be studied in this way and the faulty metabolism should be looked to as a matter of prophylaxis.

Dr. Niles said there were two phases of the paper to be mentioned particularly. He believes the wholesale indictment of tobacco in undeserved and is proven by the combined experiences of thousands of men who use the weed. As to meat, there is such a thing as a parsimony of nutrition and he called attention to the fact that history shows the races that are most successful, both as to the character of their men and the general results accomplished, were meat eaters.

Dr. Clarke commended the paper as a whole in its study and investigation, but he could not agree altogetner as to the etiology set forth. He said the Indians were the first smokers and if they did not have sclerosis how is it the cause among us? The struggle for existence and the chase after the almighty dollar is the most potent cause, not only of sclerosis but of Bright's Disease. Toxic studies are still in their infancy and we can not decide definitely what they do. The whole matter of alcohol and tobacco is subjulice.

Dr. Todd thanked Dr. Neal for his paper, but does not agree with all of his statements. He said that nature's method in repairing arterio sclerosis was lime deposits, but it is not always to be trusted; it may do too much. Treatment requires great care of the heart in not making it contract too forcibly. Kidneys are usually involved but should not be pressed upon too much. The Sphygiomanometer should always be used. Oxalate lime and its significance is frequently overlooked. Fat meats should be used in the diet and a man should not suddenly be taken from his habitual use of tobacco and whiskey.

Dr. Westmoreland said that his ideas were that arterio sclerosis is a symptom of previously existing conditions due to errors of diet. Alcohol is effective only because it influences the digestion. Physiologic rest is a part of the treatment. Where the kidneys are involved put the patient to bed and keep him there to get results. Indicanurea is only a symptom of faulty digestion and frequently there is a mechanical obstruction at the ilio coecal valve which is overlooked. The total acidity of the urine is not considered carefully enough; it should be examined and cleared away when present. Skatol if siffused is a sign of obstinate cases and accompanies high total acidity. In treatment he puts his patient to bed, gives free catharsis and light diet. Occasionally he has bled sthenic patients with success.

Dr. Strickler said Dr. Westmoreland had expressed his ideas pretty clearly. It is the man who has intestinal trouble, from whatever causes, who suffers with the disease. Business men and women have disease as much as anyone. He does not believe in intestinal antiseptics except willow charcoal. Beta napthol and napththaline are good antiseptics but not of much use in this disease. When lime salts are present in the arteriosclensio we have atheroina which is away beyond the sclerosis period. Their presence protects the vessels from undue dilation and aneurisms. If there is a low blood pressure at the time one may be sure it is subsequent to high one and due to a weak heart. Venesection if often of value.

Dr. Neall said the ductless glands tend to overcome the toxines in the system. In any acute disease where the patient has been using meat, tobacco and alcohol the prognosis is worse than if he had been abstemious. The irritation comes direct to the arteries from these substances. Going back into history he said that before the flood the men were not meat eaters and they lived several hundred years.

Dr. F. K. Boland read a paper on "Conservative Surgery in the Uterine Appendages".

Dr. Westmoreland said that in pelvic operations the psychological manifestations were not sufficiently studied. Morbidity of mind is not necessarily dependent upon the surgical operation or the removal of the organs. In the psychological thenomena after the operation other factors are often introduced. The woman has not been sufficiently educated in preparing her for the operation and she expects all sorts of things, many of which have been told to her by other women, and brings them about by her very introspection. When she has been carefully instructed that the surgical interference of the organs will in all probability produce no change of function except, that directly related to them, she usually has no trouble. This does not mean that conservatism should not guide the surgeon nor that the organs should be removed at any time unless absolutely necessary. Leaving them out of place after an operation while adhesions may occur will bring about post-operative conditions that debilitate the patient to no little degree. The ovaries being removed there is a tendency for the uterus to fall backward and it should be anchored into place as a matter of routine and if this is done there is no stress on the parts and practically no psychologic symptoms ensue. Secondary operations are advisable whenever adhesions are present and cause any trouble because it is they rather than the absence of the organs themselves which make the disturbance. Preparation for these operations should be included under physiologic rest.

Dr. Duncan said he agreed with Dr. Westmoreland as to the encouragement and training of women prior to an operation and then she should know exactly what has been done and what it meant.

Dr. Boland said, in closing, that the careful toilet in the operative field would reduce dangerous adhesions to the minimum. Careful methods of stitching (Lamberts sutures) have this result.

